# Abnormal lipid metabolism in cancer-associated cachexia and potential therapy strategy

**DOI:** 10.3389/fonc.2023.1123567

**Published:** 2023-05-02

**Authors:** Ruoxin Fang, Ling Yan, Zhengkai Liao

**Affiliations:** ^1^ Department of Radiation and Medical Oncology, Zhongnan Hospital of Wuhan University, Hubei Key Laboratory of Tumor Biological Behaviors, Hubei Cancer Clinical Study Center, Wuhan, Hubei, China; ^2^ Department of Cardiology, Renmin Hospital of Wuhan University, Hubei Key Laboratory of Metabolic and Chronic Diseases, Wuhan, Hubei, China

**Keywords:** lipid metabolism, adipose tissue, cancer, cachexia, therapy strategy

## Abstract

Cancer-associated cachexia (CAC) is a major characteristic of advanced cancer, associates with almost all types of cancer. Recent studies have found that lipopenia is an important feature of CAC, and it even occurs earlier than sarcopenia. Different types of adipose tissue are all important in the process of CAC. In CAC patients, the catabolism of white adipose tissue (WAT) is increased, leading to an increase in circulating free fatty acids (FFAs), resulting in “ lipotoxic”. At the same time, WAT also is induced by a variety of mechanisms, browning into brown adipose tissue (BAT). BAT is activated in CAC and greatly increases energy expenditure in patients. In addition, the production of lipid is reduced in CAC, and the cross-talk between adipose tissue and other systems, such as muscle tissue and immune system, also aggravates the progression of CAC. The treatment of CAC is still a vital clinical problem, and the abnormal lipid metabolism in CAC provides a new way for the treatment of CAC. In this article, we will review the mechanism of metabolic abnormalities of adipose tissue in CAC and its role in treatment.

## 1 Introduction

Cancer-associated cachexia (CAC) is a “multifactorial syndrome” characterized by increased catabolism, weight loss, and decreased skeletal muscle mass and strength (with or without adipose tissue loss) (1). CAC associates with almost all types of cancer and accounts for a quarter of cancer-related deaths (2). Prevalence of cachexia ranges 50 to 80% in advanced cancer (3). CAC is a continuum with three stages of clinical relevance: precachexia, cachexia, and refractory cachexia. Patients who have more than 5% loss of stable body weight over the past 6 months, or a body-mass index (BMI) less than 20 kg/m² and ongoing weight loss of more than 2%, or sarcopenia and ongoing weight loss of more than 2%, but have not entered the refractory stage, are classified as having cachexia (1). Early CAC can also occur in patients with curable cancer and can be reversed by appropriate treatment (4). However, we often do not diagnose CAC in cancer patients until weight loss has occurred, and there are still few methods for early diagnosis of CAC.

CAC is due to the negative energy balance metabolic changes caused by higher energy demand from the tumor and reduced calorie intake of the host, including inflammation, increased catabolism and excessive energy consumption (5–7). It can lead to multi-organ functional disturbance, which is associated with increased susceptibility to infection, higher incidence of metastasis, decreased response of cancer cells to treatment, decreased quality of life, and poor prognosis (1, 8–11). CAC is affected by endogenous and environmental factors, such as complications, genetic risk factors, gender, age, and anti-tumor treatment (12–14). It has been pointed out that tumor or host derived cytokines can affect the metabolism of CAC (15). Cachexia mainly damages skeletal muscle, adipose tissue, liver, brain, intestine, pancreas, bone, and heart (16). The metabolic disorders can further aggravate this multifactorial syndrome (17).

There are many changes in adipose tissue metabolism in cancer patients. Increasing evidence demonstrates that adipose wasting occurs before muscle loss in the early stage of CAC (18). No matter what the patient’s weight is, fat loss is an adverse prognostic factor for terminal cancer (19). Studies have shown that the changes of adipose tissue morphology and function in CAC patients have important clinical significance, preserving adipose mass and correcting abnormal lipid metabolism in CAC represents a promising therapeutic strategy (20).

However, the knowledge about mechanisms of abnormal lipid metabolism in CAC is still limited. In this review, we summarized the classification and characteristics of adipose tissue and the changes of them in patients with CAC. Finally, we described existing therapeutic approaches and discussed potential new strategies that arose by targeting the link between adipose tissue and cachexia, with a view to providing directions for future clinical treatment of CAC.

## 2 Adipose tissue

Adipose tissue is a large, interactive multi-chamber organ with clear histological and anatomical structure (21). Mature adipocytes account for only one-third of adipose tissue, and the remaining two-thirds of adipose tissue are composed of nerves, blood vessels, fibroblasts, and adipocyte precursor cells (22). It was found that adipose tissue not only played multiple and complex roles in mechanical buffering and energy storage, but also had paracrine and endocrine functions as an important secretory organ (23, 24). It can regulate energy balance and homeostasis *in vivo* from many aspects, including appetite, inflammation, insulin sensitivity, and lipid metabolism (25). Nowadays, increasing evidence shows that there are changes in lipid metabolism in patients with CAC.

According to its distribution, adipose tissue can be divided into subcutaneous adipose tissue (SCAT) and visceral adipose tissue (VAT), which have different anatomical, metabolic and endocrine characteristics. SCAT accounts for about 80% of total body fat in healthy adults (26). SCAT can be further divided into superficial SCAT and deep SCAT (27). VAT is mainly distributed in the abdominal cavity and retroperitoneum. The metabolic functions of VAT and SCAT are quite different. For example, compared with SCAT, visceral adipocytes have more active metabolism and greater lipolysis activity. Adipocytes of VAT have stronger insulin resistance than those of SCAT (28, 29). At the same time, SCAT is the main source of leptin production (26). Excess energy accumulates in adipocytes of SCAT, which acts as a metabolic pool. Visceral fat accumulation occurs only when SCAT capacity is insufficient or damaged.

According to functional characteristics, adipose tissue can be divided into three types: white adipose tissue (WAT), brown adipose tissue (BAT), or beige adipose tissue.

WAT is the most common type. White adipocytes are the main storage space of triglyceride. The main function of white adipocytes is to store fat and regulate free fatty acids (FFAs). It is mainly composed of large spherical adipocytes, in which single lipid droplets occupy the majority of the cell volume and mainly store energy in the form of triglycerides (21). WAT exists in subcutaneous and visceral tissues, and the increase of WAT quality in viscera is associated with increased metabolic risk (27, 30). WAT has important endocrine and paracrine effects (21, 31, 32).

Unlike WAT, brown adipocytes in BAT contain a large number of mitochondria and scattered lipid droplets. The main function of BAT is energy dissipation, which provides non-shivering thermogenesis to the body during energy-demanding conditions such as exercise, fasting or cold stimulus (33). BAT is mainly located in the interscapular region and perirenal regions of rodents and infants (34, 35), but BAT is normal component of several subcutaneous and visceral depots and is not exclusive to these areas (21). The development and gene characteristics of WAT and BAT are different (36–38). Classic brown adipocytes come from myogenic factor 5 (Myf-5) cell lines, while white adipocytes come from non Myf-5 cell lines (39). Thus, brown adipocytes are labeled with Myf-5 (40) and paired box 7 (41), similar to myogenic precursor cells. BAT contains rich vascular tree and dense capillary network (25). BAT consumes energy in the form of heat production (42–44), which is mainly due to the high level expression of uncoupling protein 1 (UCP1) in mitochondria and its proton leakage pathway (45–47), which is vital to lipid oxidation and thermogenesis. BAT, as an endocrine organ, regulates energy homeostasis by consuming fatty acids and glucose, and plays a key role in carbohydrate and lipid metabolism (47, 48).

Thirdly, there is a type of adipocyte, defined as “Brite” (white brown) (49) or “Beige” (50), which is derived from pre-existing white adipocytes (21). Beige adipocytes have plasticity and can be transformed from WAT by a variety of different pathways (21, 51, 52). The function of beige adipocytes is similar to that of brown adipocytes (49). In patients with CAC, beige adipocytes can develop, expand and activate under multiple environmental stimulation, which is the target of endocrine and paracrine stimulation (53, 54). The formation of beige adipocytes can be triggered by inflammatory mediators (such as interleukin-6 [IL-6] (55)) and tumor-derived compounds (such as parathyroid hormone related protein [PTHrP] (56, 57)). In mouse models, the formation of most beige adipocytes is a strong response to environmental factors, such as long-term low-temperature exposure (35).

The genetic characteristics of brown adipocytes and beige adipocytes partially overlap, except for specific markers, such as zinc finger (Zic1) in cerebellum (58). The common characteristics of brown and beige adipocytes are a large number of lipid droplets and dense accumulation of UCP1 positive mitochondria, although brown adipocytes had higher UCP1 expression level (39). Although brown and beige adipocytes are similar in morphology and biochemistry, they also have some distinct characteristics (35), because they come from different embryonic precursor cells (40). For example, brown adipocytes are mainly located in the interscapular and perirenal regions of rodents, while beige adipocytes exist in various WAT pools, especially in inguinal subcutaneous adipose tissue (59).

All three types of adipose tissue are important in the energy balance disrupted by CAC. WAT and BAT usually have opposite physiological functions. WAT is responsible for energy accumulation of lipid droplets in cells, while BAT is responsible for energy dissipation through heat production. Clinically, browning of WAT and activation of BAT are effective methods to combat obesity and metabolic syndrome, but in CAC we may need to block these mechanisms in order to preserve more adipose tissue. Changes in lipid metabolism under local or systemic stimulation make it a potential cause of CAC. In the case of congenital or acquired lipodystrophy, cachexia or any other severe malnutrition, there is almost total lack of adipose tissue, even severe multiple organ dysfunction results from the lack of leptin and other adipokines.

## 3 Changes of lipid metabolism in patients with CAC

Lipid metabolism and adipose tissue mass are regulated by two pathways: lipolysis and lipogenesis. Lipolysis and lipogenesis balance maintain the dynamic balance of adipocytes and regulate the energy balance of CAC patients. Adipose tissue atrophy in cancer patients is attributed to increased lipolysis and lipid oxidation, decreased lipogenesis, impaired fat deposition and lipogenesis, and browning of WAT (60). Compared with non-cancer patients, the volume of adipocytes in cancer patients was smaller, but the total number of adipocytes did not change. Adipocytes isolated from patients with cachexia showed stronger catecholamine and natriuretic peptide-induced lipolysis (61). Weight loss patients also showed more sensitive characteristics to catecholamine signal (62). In addition, compared with cancer patients without cachexia, the expression of UCP1 in adipose tissue of CAC patients is higher, which may lead to adipose tissue atrophy and more heat production (55).

Patients with CAC often show systemic hypermetabolism with reduced energy intake and increased energy consumption, especially the abnormal increase of resting energy expenditure is considered to be the main cause of energy consumption. CAC, which is characterized by adipose tissue loss (60), is the terminal manifestation of metabolic changes in adipose tissue. The metabolic changes of adipose tissue have been proved to play an important role in CAC. In the context of CAC, changes in lipid metabolism and energy consumption have been shown to be harmful (16). In patients with terminal cancer, reduced adipose tissue is associated with poor prognosis (18, 19). In cachexia, fat loss is faster and earlier than lean tissue loss, especially in the period before death.

**Figure 1:**
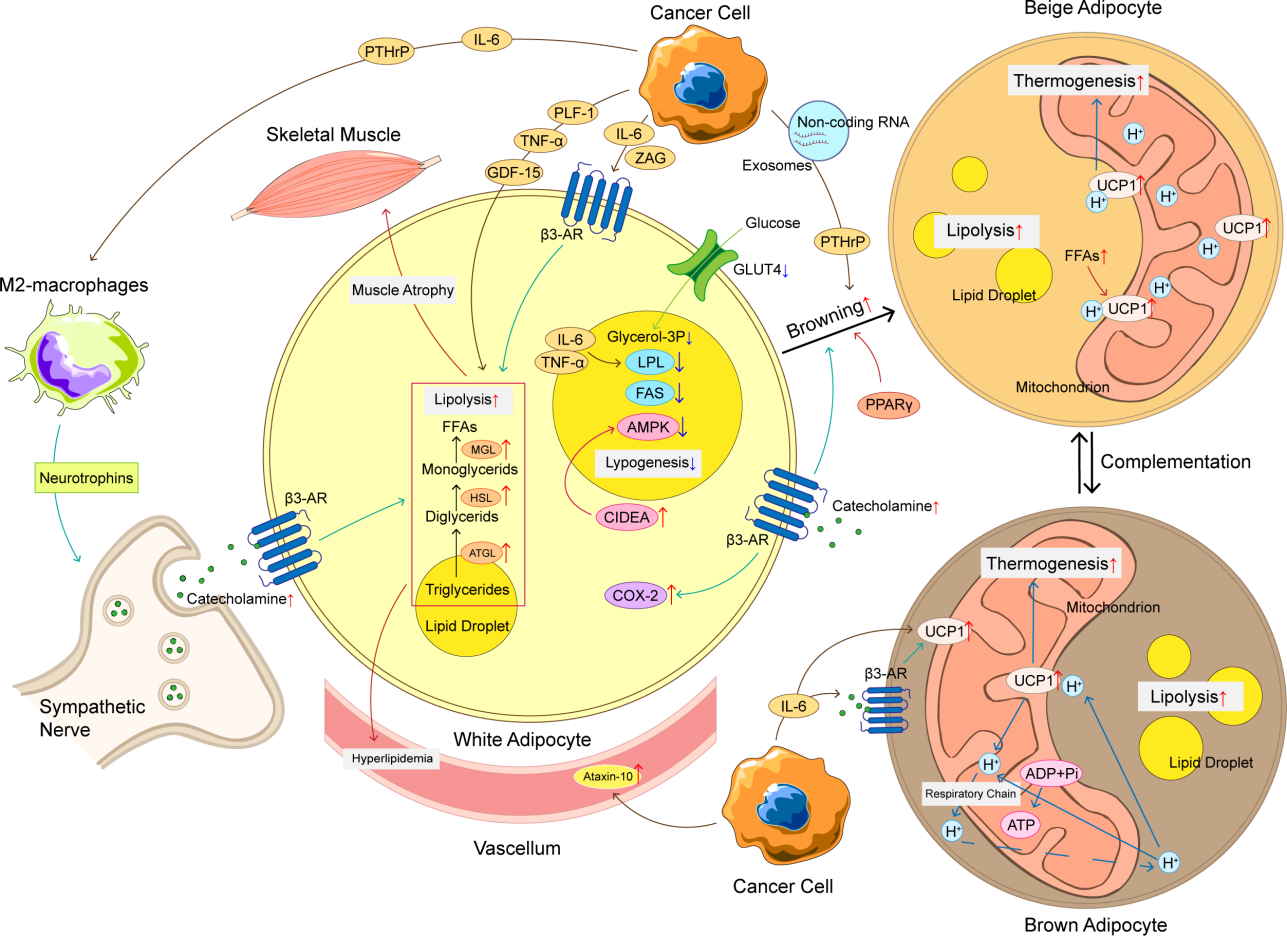
The mechanism of adipose tissue loss in CAC. The mechanism of adipose tissue loss in CAC is complicated, and these mechanisms can be roughly divided into increased lipolysis, browning of WAT, increased thermogenesis of BAT, and decreased lipogenesis. Cancer cells produce a variety of cytokines to promote adipose tissue loss, such as IL-6, TNF-α, PTHrP, etc. These cytokines not only promote lipolysis, but also promote WAT browning and upregulation of UCP1 in BAT, ultimately leading to an increase in thermogenesis and energy consumption. At the same time, the crosstalk between adipocyte and immune cells and nervous system also plays an important role in abnormal lipid metabolism of CAC. The loss of adipose tissue has a chain reaction that can produce hyperlipidemia and promote skeletal muscle atrophy, making CAC patients’ condition worse. CAC, cancer-associated cachexia; WAT, white adipose tissue; BAT, brown adipose tissue; FFAs, free fat acids; ATGL, adipose triglyceride lipase; HSL, hormone-sensitive lipase; MGL, monoglyceride lipase; LPL, lipoprotein lipase; FAS, fatty acid synthase; UCP1, uncoupling protein 1; β3-AR, adrenoceptor beta 3; ADP, adenosine diphosphate; ATP, adenosine triphosphate; IL-6, interleukin-6; TNF-α, tumor necrosis factor α; PTHrP, parathyroid hormone related protein; ZAG, zinc-α 2-glycoprotein; PLF-1, proliferin-1; GDF15, growth differentiation factor 15; CIDEA, DNA fragmentation factor like effector A; AMPK, adenosine 5’-monophosphate (AMP)-dependent protein kinase; COX-2, cyclooxygenase 2; GLUT-4, glucose transporter 4.

Adipose tissue contributes to weight loss in starvation, while skeletal muscle and adipose tissue mass decrease significantly in CAC patients. Lipolysis of adipocytes is the main cause of adipose tissue loss in cancer patients, and plays an important role in the course of CAC, and it is not related to malnutrition (63). The mechanism of adipose tissue loss in CAC is believed to be due to increased lipolytic activity and lipid utilization (64), while other mechanisms, such as impaired lipogenesis, may also be one of the reasons (**Figure 1**). In the experimental model of cachexia, the decrease of adipose tissue appeared before the decrease of skeletal muscle mass and food intake (65). Studies have also shown that the decrease in adipose tissue is due to a significant decrease in adipocyte size resulting from a decrease in fat reserve, rather than a decrease in the number of cells (cell death) (64, 66). Some studies have found that lipogenesis and lipoprotein lipase (LPL) expression and activity have not been significantly down regulated in CAC patients (67, 68). A study identified the marker components of “cachectin” including Ataxin-10, which are sufficient to trigger abnormal fatty acid metabolism and cardiac atrophy. The serum level of Ataxin-10 is significantly increased in CAC patients (69).

### 3.1 Catabolism of WAT

The reduction of WAT in visceral and subcutaneous tissues plays an important role in CAC (60). High lipolysis is an important feature of cachexia in cancer patients and rodents (70, 71). Lipolysis depends on three kinds of lipases: adipose triglyceride lipase (ATGL), hormone-sensitive lipase (HSL) and monoglyceride lipase (MGL). Increased expression and activity of these three enzymes lead to the decrease of adipose tissue and the increase of circulating FFAs and glycerol (68, 72).

In CAC patients, the high levels of circulating FFAs and glycerin is caused by the involvement of ATGL and HSL in the catabolism of triglyceride in WAT (73). Excess free lipid molecules are “lipotoxic”, leading to cellular dysfunction and even death, insulin resistance in animals and humans, and have side effects on many organs (16, 74, 75). Compared with cancer patients with stable weight, the expression of HSL was increased in patients with CAC (61, 76). A large amount of evidence shows that the overexpression of ATGL and HSL in WAT of CAC patients is related to the decrease of BMI (73). Inhibition of HSL or ATGL expression in mice not only retained WAT, but also reduced the loss of skeletal muscle, which indicated that mutual regulation between adipose tissue and muscle play an important role in CAC (71, 73). CGI-58 can bind and activate ATGL (ATGL is the only lipase activated by CGI-58) (77). G0S2 (G0/S2 switch gene 2) is considered to be the main selective inhibitor of ATGL, which can weaken the effect of ATGL *in vitro* and *in vivo*, and regulate triglyceride hydrolysis through this mechanism (78).

In addition, patients with cachexia often have insulin resistance or decreased insulin secretion (79), which may be due to the fact that the body prevents insulin from playing its anti-lipolysis role in CAC. Transcriptome analysis of adipose tissue from patients with gastrointestinal cancer cachexia showed that the expression of related pathways regulating energy conversion was up-regulated (64). Both tumor cells and host immune cells (such as macrophages and lymphocytes) release cytokines or hormones, such as IL-6, tumor necrosis factor α (TNF-α), zinc-α 2-glycoprotein (ZAG, also known as lipid mobilization factor), proliferin-1, catecholamines, and natriuretic peptide, which can promote lipolysis and reduce insulin sensitivity in CAC patients (6, 73, 80, 81). They are involved in the regulation of proinflammatory state, stress response, anorexia, disease behavior, hypermetabolism and accelerated decomposition of protein, muscle and adipose tissue in CAC patients (82). Systemic inflammation and changes in the human immune system are important determinants of this state (83, 84), which suggest that inflammatory cytokines may be biomarkers for the diagnosis of CAC (71).

### 3.2 Adipose tissue browning

In mammals, adaptive thermogenesis occurs mainly in brown and beige adipocytes (35). The increase of energy consumption can also be explained by the increased heat production and “browning” of WAT (55, 56). WAT browning refers to the transformation from WAT to BAT, and its name comes from the dark color associated with mitochondria. The gradual transformation of adipose tissue types is an interesting feature of CAC.

This process promotes mitochondrial respiration, leading to thermogenesis rather than ATP synthesis, thus activating lipid mobilization and increasing energy consumption (85). Beige adipocytes, which are phenotypically different from those in WAT and BAT, can appear under severe cold exposure (86), adrenergic stimulation (87) and prostaglandin synthase (cyclooxygenase 2, COX-2) (88). These cells can significantly promote total energy consumption and lead to fat loss (89). Obesity related studies have shown that the central nervous system, especially the hypothalamus, is an important regulatory organ for browning (87). Klaus Felix et al. found that the level of glucagon-like peptide-1 (GLP-1) was increased in pancreatic cancer cachexia patients (90), GLP-1 appears to trigger satiety and inhibit food intake through molecular regulation in the hypothalamus (91). At the molecular level, transcription factors such as peroxisome proliferator activated receptor gamma (PPARγ), PPARγ coactivator 1α (PGC-1α), transcription factor PR domain-containing 16 (PDRM16) and CCAAT enhancer binding protein β (C/EBP-β) regulate the transcription events of differentiation into brown adipocytes (92, 93). This is related to upregulation of UCP1 expression, increased heat production (non-shivering), loss of ATP production and increased energy catabolism (73). WAT atrophy and adipose tissue browning occurred in the early stage of CAC (94). The loss of brown adipocytes may lead to browning of WAT, which indicates that there is a compensatory mechanism between mature brown adipocytes and beige adipocytes (95).

In adipose tissue, proinflammatory factors promote the browning of WAT. With the development of CAC, inflammatory microenvironment and metabolic disorder caused by IL-6, TNF-α and parathyroid related peptide secreted by tumors and host immune system will promote browning of WAT (59, 94). IL-6 promotes systemic metabolism to a certain extent by regulating BAT activation and adipose tissue browning (80). In addition, tumor-derived IL-6 and adrenoceptor beta 3 (β3-AR) activation is associated with CAC mediated adipose tissue browning. Neutralization of IL-6 or β3-AR can significantly improve cachexia (55). The introduction of IL-6 into the brain by adenovirus vector can significantly increase the expression of UCP1 in sympathetic innervated brown adipocytes, but not in denervated brown adipocytes, indicating that IL-6 might activate BAT through β3-AR signaling pathway (96). Although PTHrP treatment did not change tumor size in Lewis mouse lung CAC model, it resulted in CAC related weight loss and skeletal muscle atrophy, and activated beige cells to produce heat. On the contrary, blocking PTHrP with neutralizing antibody can prevent adipose tissue and skeletal muscle atrophy. In addition, PTHrP shares the G-protein coupled receptor signaling pathway with β 3-AR agonists, and upregulates the protein expression level of UCP1 in white and brown adipocytes (56). Therefore, tumor cell-derived IL-6 and PTHrP may play an important role in CAC by activating BAT and/or adipose tissue browning.

Two G-protein coupled receptors, called FFA receptor 1 (FFA1) and FFA receptor 4 (FFA4), were identified as molecular targets for ω-3 polyunsaturated fatty acids (97, 98). When activated, these receptors can promote a variety of effects, such as increasing insulin sensitivity, inducing adipose tissue browning, promoting analgesia by releasing β-endorphin, controlling energy homeostasis, and reducing food intake (99, 100). Activation of FFA4 results in browning of adipose tissue (99, 101). Lewis lung cancer mouse models lacking the adipose tissue specific PRDM16 showed reduced browning, thermogenesis and lipoatrophy (56). By neutralizing browning promoting hormones, such as PTHrP, the improvement of CAC and the reduction of adipose tissue loss were observed in animal models (56). WAT showed heterogeneity in browning efficiency. Some parts such as visceral adipose tissue is resistant to browning. It has been reported that visceral adipose tissue browning may be a compensatory heat production mechanism (102), but its conversion mechanism is still unclear. Hongmei Yang et al. found that exosomes from Lewis lung carcinoma cells can induce lipolysis *in vitro* and *in vivo* by delivering PTHrP, and inhibition of exosome generation prevented the fat loss of tumor bearing mice (103, 104). Furthermore, there have been many studies in recent years confirming that non-coding RNA plays an important role in the browning of WAT. Wenjuan Di et al. found that miR-146b-5p was enriched in cancer-related exosomes, which plays an essential role in WAT browning. miR-146b-5p can directly repress the downstream gene homeodomain‐containing gene C10 (HOXC10), thereby regulating lipolysis (105). Non-coding RNA such as miR-155, miR-425-3p, and miR-182-5p have also been shown to play a role in promoting WAT catabolism and browning in several cancer species (106–108). Further study on the mechanism of systemic metabolic and inflammatory changes leading to the transformation of WAT into BAT can further improve our understanding and treatment of cachexia.

### 3.3 Activation of BAT

There is a lot of evidence that BAT is activated under different conditions of cachexia. The enhanced heat production of BAT is considered to be one of the main reasons for the increase of resting energy expenditure in cancer patients (72). The activity of BAT was also positively correlated with the stage of cancer (109).

BAT is characterized by high mitochondrial content and increased expression of UCP1. UCP1 regulates body temperature through oxidative phosphorylation of uncoupling ATP, resulting in increased energy consumption, increased heat production, and lipolysis, leading to weight loss and progression of CAC (56, 59, 94). The activation of BAT is mediated by β3-AR, which is activated by the sympathetic nervous system, leading to adipocyte contraction (56). β3-AR was activated and UCP1 expression was increased, which activated the delipidation in BAT (110). Catecholamine signaling in BAT transduction was enhanced in cachexia mice, but blocking the β3-AR by propranolol could prevent the increase of body temperature (111). Catecholamine levels are associated with BAT activity and BMI (112). Moreover, FFAs released by lipolysis are direct activators of UCP1 (33), indicating that enhanced WAT catabolism in CAC will promote BAT activation.

Brown adipose precursor cells expressing early B-cell factor 2 and platelet-derived growth factor receptor α differentiated into mature brown adipocytes (113). The regulation of BAT depends on a variety of cytokines. β1-adrenergic receptor (ADRB1) mediates norepinephrine induced BAT formation (114). The expression of ADRB1 was correlated with the rate of lipolysis in patients with CAC (76), Prep1 is a adipo-osteogenesis regulatory factor, which is related to the increase of BAT density and osteogenesis reduction (115). IL-6 also plays an important role in mediating BAT activation by increasing the expression of UCP1 and activating fatty acid β-oxidation related gene thermogenesis in gastric cancer and colon cancer patients with cachexia (80). With Lewis lung cancer model, it has been proved that tumor-derived PTHrP regulates the expression of genes involved in adipose tissue thermogenesis, lipolytic enzymes and muscle atrophy. These studies suggest that blocking BAT activation may be a way to treat cachexia. However, due to the difficulty of BAT localization in human body, the research progress on BAT is very slow at present.

### 3.4 Crosstalk between adipocyte and non-adipocyte

In the context of CAC, there are communications between adipocytes and a variety of non-adipocytes, which is the key to control energy homeostasis and prevent metabolic diseases. DNA fragmentation factor like effector A (CIDEA) is an important metabolic regulator and apoptosis inducing factor. Adipocyte dysfunction leads to the increase of CIDEA, followed by the degradation of adenosine 5’-monophosphate (AMP)-dependent protein kinase (AMPK) in cachexia adipose tissue (116). By hypothalamic AMPK signal, the secretion of leptin, adiponectin and insulin are controlled (117). When the effect of insulin is enhanced, IL-6 secreted by skeletal muscle will increase. IL-6 acts on muscle contraction also by activating AMPK (117). Meanwhile, IL-6 induces Toll-like receptor-4 gene expression via activation of STAT3, leading to insulin resistance in human skeletal muscle, which further accelerates muscle wasting (118). Lipolysis results in increased FFAs in circulation, and FFAs will eventually enter skeletal muscle and cause muscle atrophy (91). Blocking fatty acid oxidation not only rescued human myotubes, but also improved muscle mass and body weight in CAC models *in vivo*, which indicates that there may be interaction between adipose tissue and skeletal muscle (119). Rowena Suriben’s study has indicated that growth differentiation factor 15 (GDF15) elicits a lipolytic response in adipose tissue and leads to reduced adipose and muscle mass and function in tumor-bearing mice, inhibiting GDF15-driven lipid mobilization and oxidation can be translated to preservation of skeletal muscle mass (120). GDF15 regulates survival of motor and chipmaker sensory neurons (121), so lipid and skeletal muscle may interact through neurons.

During cachexia, systemic inflammation is one of the main driving forces of fat consumption. Cancer cells secrete a variety of mediators, such as TNF-α, IL-6, IFN-γ, ZAG and PTHrP, which can promote browning (55, 56, 122). TNF-α belongs to cachectin (123), which can be released from adipose tissue and mediates CAC by reducing the expression of glucose transporter 4 (GLUT4), which in turn inhibits glucose transport and adipogenesis (124). In addition, adipose tissue is closely related to inflammatory cells, the cross-talk between immune response and adipose tissue biology has been proved. Macrophages can infiltrate WAT and activate immune responses in cachexia mice. A study has shown that macrophages can regulate WAT browning through paracrine heat shock protein A12A (125). Recently, Hao Xie et al. demonstrated that the immune-sympathetic neutron communication axis is essential for WAT browning in CAC. IL-6 and PTHrP can activate immune cells, including macrophages. Then, the type 2 macrophages will produce neurotrophins and increase polyamine synthesis and secretion. This increases neuronal activity, leading to enhanced local catecholamine synthesis, β-adrenergic stimulation of WAT, and WAT browning (126). Not only that, virus-specific CD8(+) T cells caused morphologic and molecular changes in adipose tissue, which may also lead to cachexia (127). Another study implicates adipocytes as predominantly negative regulators of the surrounding myeloid cells (128). At the same time, studies have shown that the imbalance of the immune system affects the gut microbiota (129), which also plays an important role in CAC. One clinical study has shown that compared with non-cachexic people, *Proteobacteria*, an unknown genus from the *Enterobacteriaceae* family, and *Veillonella* were more abundant among CAC patients (130). Currently, the crosstalk between adipose tissue and other tissues is attracting more and more attention, and the study of these mechanisms may provide us with a more holistic understanding of CAC, so as to develop diagnosis and treatment strategies.

### 3.5 Reduced lipogenesis

The decrease of fat mass in cancer patients depends on the decrease of lipid deposition and lipogenesis. In the process of lipogenesis in adipose tissue, some fatty acids will be re-esterified to triglycerides, forming a futile cycle, which is mediated by AMPK pathway, and the activity of AMPK pathway in cachexia adipose tissue is decreased (116). The activities of fatty acid synthase (FAS) and LPL in adipose tissue of cancer patients were decreased (131). A large number of animal studies have also shown that the activity of LPL is reduced in cancer (132). LPL hydrolyses free circulating triacylglycerol present in chylomicrons very-low-density lipoproteins (VLDLs), whose decreased activity is associated with increased IL-6 (133). Compared with cancer patients with stable weight, patients with CAC have more adipose tissue oxidation (134). Mice-bearing colon adenocarcinoma showed an increase in LPL activity in the heart and adipose tissue increasing weight loss but decreasing with further weight loss. It is suggested that the initial rise in LPL activity provides more oxidation of fatty acids in cachexia state (7, 135). These evidences suggest that the dysfunction of LPL is related to the occurrence and development of CAC, and testing the activity of LPL may be useful for the diagnosis of cachexia. In the experimental model of CAC, the process of lipogenesis was weakened and the expression of lipogenic transcription factors was decreased, which was related to the decrease of adipocyte size and the higher expression of TNF-α (136–138). Besides, non-coding RNA also affects lipogenesis, Diya Sun et al. found that the expression of miR-410-3p was higher in subcutaneous adipose tissues and serum exosomes of CAC patients, which significantly inhibited adipogenesis and lipid accumulation (139). Additionally, due to anorexia associated with CAC or the difficulty of eating due to cancer, the intake of lipids and other nutrients will be reduced, which will also lead to the reduction of lipogenesis.

## 4 Therapy strategy

However, the research progress in the treatment of CAC and the improvement of patients’ prognosis is relatively slow, there is also no definite treatment plan for the abnormal lipid metabolism of CAC patients. Studies have shown that CAC is often reversible if intervention is carried out in pre-cachexia or cachexia stage (10, 140). The main treatment strategies used in these stages include exercise and nutritional support, as well as the elimination of any direct cause of cancer (such as reduced food intake or malnutrition due to obstruction or compression) (140, 141). Unfortunately, many cases have been diagnosed as refractory cachexia, when CAC is usually irreversible (1, 140). At present, the main researches focus on the treatment of CAC. Several treatment options have been proposed, but have not been clinically confirmed. The treatment combination mainly involves two pathways: the anabolic pathway and the antimetabolic pathway for muscle and fat catabolism (82). Neutralization of metabolic changes is the first task to overcome cachexia, including the control of skeletal muscle protein decomposition rate, as well as the control of liver, lipid and carbohydrate protein metabolism abnormalities.

A retrospective study by Dingemans et al. included 12 phase II clinical trials involving 11 compounds. These drugs fight CAC through one of the following mechanisms: increase appetite, improve digestion, reduce systemic inflammation, and increase the ratio of muscle synthesis and degradation (142). Many other drugs have entered phase III trials, but it is difficult to achieve multiple clinical endpoints at the same time. Anamorelin, an auxin receptor agonist, has demonstrated its ability to improve lean weight in phase II clinical trials in patients with non-small cell lung cancer and CAC (143). However, the results of this phase II trial and subsequent phase III trial showed that although lean weight was improved, grip strength did not improve (143, 144), which was rejected by the European Drug Administration in 2017. Enobosarm, a selective androgen receptor regulator, showed a significant increase in total lean body weight in the phase II study (145), but it did not produce consistent end-point results in the phase III trial (146, 147). There are many influencing factors in CAC. Current studies cannot fully elucidate the pathophysiological mechanisms of CAC, so as to effectively reduce or reverse all clinical factors of CAC. Therefore, even if the development of many drugs is moving in the right direction, few of them can pass phase II and III studies.

As we mentioned above, lipopenia is an important feature of CAC, and several studies have suggested potential therapeutic strategies for lipid metabolism in CAC, which are summarized in **Table 1**. Our summary shows that although so many potential strategies have been identified, they are still far from clinical practice. Based on the available evidence, nutritional strategies such as supplementing patients with unsaturated fatty acids and marine phospholipids may be effective (7, 164). Some chemotherapeutic agents, such as cytarabine, promote fat depletion, and we can intervene early in the treatment of patients using these chemotherapeutic agents (165). Exercise training may also reduce inflammation and improve the condition of muscle and adipose tissue in CAC patients (166). What’s more, the application of some new technologies may be helpful to this field. The appearance of 3D bioprinted WAT model is helpful for us to further study and understand the mechanism of adipose tissue metabolism in cachexia (167). Portable biosensors can make it easier for us to monitor lipolysis in patients (168). However, these strategies still need more clinical data to support, the treatment of CAC is still a major challenge for clinicians.

**Table 1:** Potential therapeutic strategies for lipopenia in CAC.

Therapy strategy	Object	Evidence	Reference
Exercise training	Patients with cancer	Aerobic and resistance exercise can improve patients’ muscle strength and decrease the levels of TNF-α and CRP.	(148, 149)
NSAIDs	Patients with CAC	A pilot study shows patients who received celecoxib experienced statistically significant increases in weight and BMI over controls, NSAIDs may improve weight in CAC patients.	(150, 151)
Unsaturated fatty acid	Patients with digestive system neoplasm	The plasma levels of unsaturated fatty acids were decreased in patients with cachexia. Supplementation with omega-3 fatty acids significantly increased skeletal muscle mass and decreased IL-6 and TNF-α in patients.	(152–154)
Enteral feeding	Patients with CAC	Enteral feeding is associated with improvement in decreasing body fat mass and inflammatory markers (CRP) and increasing in lean body mass.	(155)
Ghrelin	Cancer patients with anorexia	Ghrelin increases the energy intake of cancer patients with anorexia. It’s found to have a predominantly positive effect on growth hormone plasma levels, weight gain, increases in lean mass, and reductions in loss of adipose tissue.	(156, 157)
Megestrol	Patients with CAC	High dose megestrol can significantly improve the appetite and body weight of some cancer patients with cachexia, especially the body fat mass.	(158)
Gut microbiome	Patients with CAC	There are differences in gut microbiota between CAC patients and non-cachexic people, however, in one prospective study, fecal microbiota transplantation is reported to be negative.	(130, 155)
Anti-diabetic agents	*In vitro* model and murine model	Metformin can deactivate HSL and counteract TNF-α induced lipolysis thereby increasing lipid synthesis and decreasing WAT browning. Rosiglitazone is able to rescue breast cell induced lipid accumulation.	(159, 160)
Lipid lowering agents	Rat model of CAC	Simvastatin attenuates loss of body weight as well as muscle mass and improves cardiac function.	(161)
AMPK-stabilizing peptide (ACIP)	*In vitro* model and tumor-bearing murine model	ACIP is able to ameliorate WAT wasting *in vitro* and *in vivo* by shielding the Cidea-targeted interaction surface on AMPK.	(116)
Carnosol	*In vitro* model and tumor-bearing murine model	Carnosol and its analogues exhibits anti-cachexia effects mainly by inhibiting TNF-α/NF-κB pathway and decreasing muscle and adipose tissue loss.	(162)
Piceatannol	*In vitro* model and tumor-bearing murine model	Piceatannol can modulate the stability of lipolytic proteins, protect tumor-bearing mice against weight-loss in early stage in CAC through preserving adipose tissue.	(20)
Farrerol	*In vitro* model	Farrerol attenuates TNF-α-induced lipolysis and increases adipogenic differentiation in 3T3-L1 cells.	(163)
ESM	Murine model	ESM supplementation ameliorates anorexia, lean fat tissue mass, skeletal muscle wasting, reduced physical function, lipid metabolism and microbial dysbiosis.	(129)
Anti-PTHrP antibody	*In vitro* model and tumor-bearing murine model	Neutralization of PTHrP in tumor-bearing mice blocks adipose tissue browning and also loss of muscle mass and strength. It also prevents the lipolytic effects of extracellular vesicles.	(56, 104)
Anti-IL-6 receptor antibody	Murine model	Anti-IL-6 receptor antibody can inhibit WAT lipolysis and browning in cachectic mice.	(80)
Selective β3-AR antagonist	Tumor-bearing murine model	Treating mice with the selective β3-AR antagonist ameliorates cachexia and decreases UCP1 levels in subcutaneous WAT.	(55)
Anti-GDF15-GFRAL antibody (3P10)	Tumor-bearing murine model	3P10 targets GFRAL and inhibits RET signaling by preventing the GDF15-driven interaction of RET with GFRAL on the cell surface. Treatment with 3P10 reverses excessive lipid oxidation in tumor-bearing mice and prevents CAC, even under calorie-restricted conditions.	(120)

TNF-α, tumor necrosis factor-α; NSAIDs, non-steroidal anti-inflammatory drugs; CAC, cancer-associated cachexia; IL-6, interleukin-6; CRP, C-reactive-protein; HSL, hormone-sensitive lipase; WAT, white adipose tissue; AMPK, adenosine 5’-monophosphate (AMP)-dependent protein kinase; NF-κB, nuclear factor kappa-B; ESM, eggshell membrane; PTHrP, parathyroid hormone related protein; β3-AR, adrenoceptor beta 3; UCP1, uncoupling protein 1; GDF15, growth differentiation factor 15; GFRAL, GDNF family receptor alpha like; RET, ret proto-oncogene.

## 5 Conclusions

CAC is a chronic disease involving multiple organs and tissues, which requires multi-mode treatment, including drug treatment, nutritional support and physical exercise, so as to better adapt to the complex mechanisms of body consumption. While enhancing the balance of anabolism/catabolism, it can improve the physical condition and improve the quality of life (169, 170). However, the ideal drugs against CAC are still under development.

**Figure 2:**
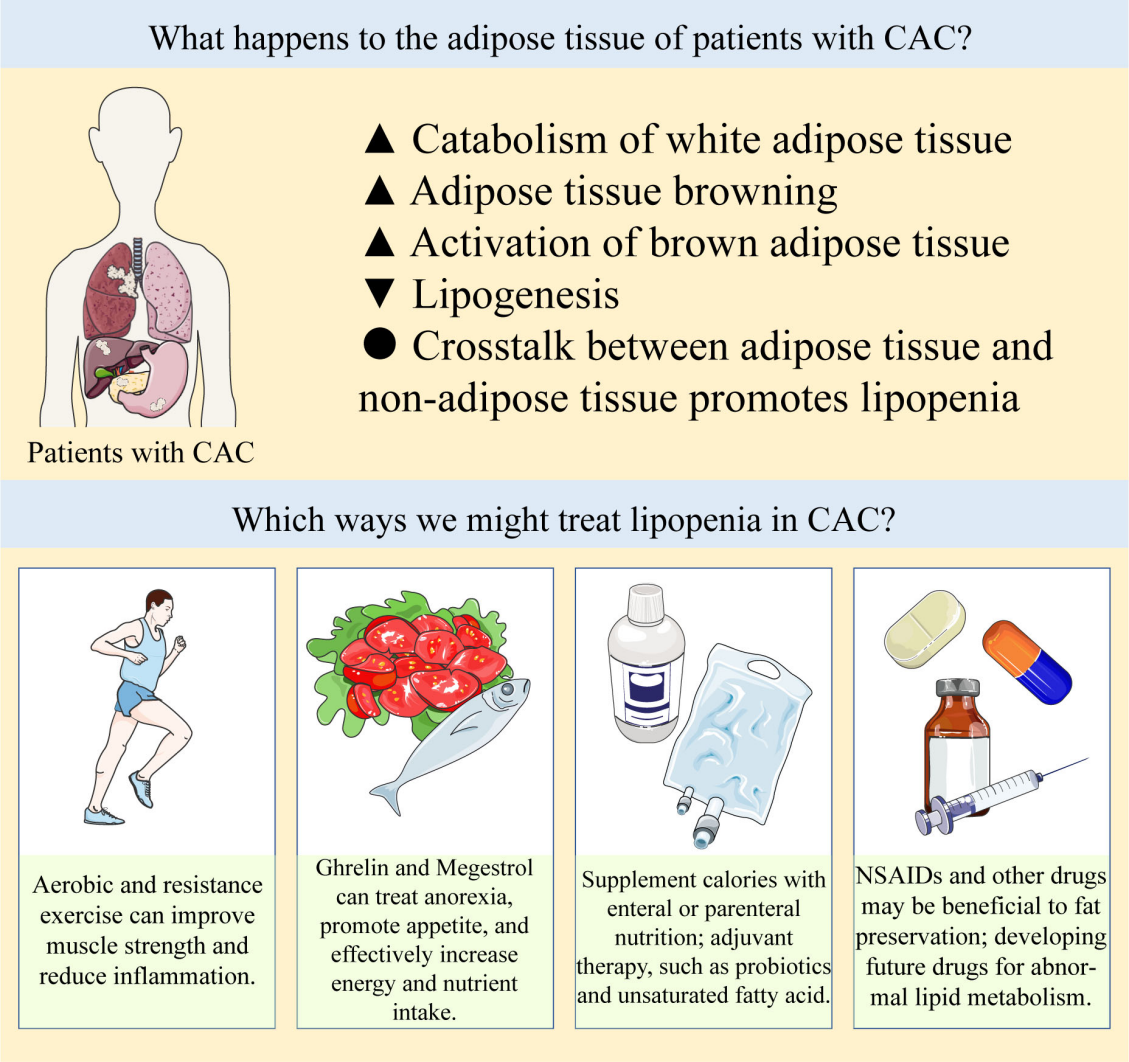
Abnormal lipid metabolism in CAC and potential therapeutic strategies.

Studies have shown that adipose tissue is involved in the formation of CAC, and abnormal lipid metabolism plays an important role in the development of CAC (**Figure 2**). In adipose tissue, WAT guides the system energy production through the balance between lipogenesis and lipolysis. BAT has been found to have important physiological and pathological functions in adults. Browning stimulates the differentiation and thermogenesis of beige adipocytes. These adipose tissues contribute differently to CAC. In the development of CAC, adipose tissue interacts with other cells or organs, showing therapeutic potential. Since abnormal lipid metabolism occurs at the early stage of CAC, correcting abnormal lipid metabolism and appropriately increasing adipose tissue may delay or even prevent the further deterioration of CAC. Therefore, it is also very important to explore related biomarkers to monitor lipid metabolism in cancer patients. In this field, we need to continue to pay attention to and think about the following questions: (1) At present, there is a lack of clinical monitoring indicators of lipid metabolism, and it is difficult for us to identify abnormal fat loss in cancer patients at an early stage. At the same time, the specific changes of each fat depot during fat loss are still not clear. (2) Some studies have shown that appropriate diet control and exercise can increase muscle strength and promote metabolic health in cancer patients. However, there is still a lack of relevant research conclusions on whether these measures are safe for patients with CAC and whether these interventions should be implemented in patients with CAC. (3) The related side effects of anti-tumor therapy may also promote the development of CAC. The effects of these therapies, including chemotherapy, radiotherapy, immunotherapy and targeted therapy, on lipid metabolism remain unclear. (4) Current animal models of CAC may not adequately simulate the actual physiological changes of CAC, many mechanistic details of abnormal lipid metabolism in CAC need to be further explored, and the interaction between different types of cancers and adipose tissue may also be different.

In this review, we systematically summarize the currently known mechanisms related to abnormal lipid metabolism in CAC. Although we have summarized a number of potential treatments for abnormal lipid metabolism in CAC in this review, most of them still require further drug development and clinical validation. CAC is still an unavoidable problem for oncologists. Through this review of abnormal lipid metabolism in CAC, we hope that drugs targeting abnormal lipid metabolism in CAC can be developed in the future, so as to effectively treat CAC and improve the prognosis of patients with CAC.

Abnormal lipid metabolism in CAC can be summarized as increased catabolism and decreased synthesis of white adipose tissue, increased browning of adipose tissue and abnormal activation of brown adipose tissue, and crosstalk between adipose tissue and non-adipose tissue further promotes adipose loss. There are a number of treatments available to prevent lipopenia, such as exercise, appetite promotion, caloric and nutrient supplementation by oral or parenteral nutrition, and NSAIDs and other drugs that may be beneficial to fat preservation, the specific items are listed in **Table 1**. In clinical practice, these strategies are often used in combination to prevent the progression of CAC, and drugs targeting the above abnormal lipid metabolism need to be developed and explored in the future.

## Author contributions

RF: Writing—Original Draft Preparation; LY, ZL: Supervision and writing — Review and Editing. All authors contributed to the article and approved the submitted version.
